# A retrospective study on pediatric pertussis: comprehensive evaluation of symptoms, laboratory indicators, and diagnostic efficacy of case definitions

**DOI:** 10.3389/fcimb.2025.1694670

**Published:** 2025-12-17

**Authors:** Junfei Guo, Weiming Lai, Qiongdan Mai, Yasha Luo, Minling Zheng, Yanting Qin, Jiana Xiong, Weixiang Wu, Mingyong Luo

**Affiliations:** 1 Department of Clinical Laboratory, Guangdong Women and Children Hospital, Guangzhou, China; 2 Guangzhou Medical University, Guangzhou, China; 3 Women and Children’s Hospital, Southern University of Science and Technology, Shenzhen, China

**Keywords:** children, China, epidemiological features, pertussis, procalcitonin

## Abstract

**Objective:**

This study aimed to characterize the epidemiology of pertussis in children, evaluate the diagnostic performance of clinical and laboratory features, assess the effectiveness of different suspected-case criteria, and identify independent risk factors for ICU admission.

**Methods:**

We retrospectively analyzed demographic, clinical, and laboratory data from patients aged ≤14 years who underwent pertussis testing. Participants were stratified by test results, and the sensitivity and specificity of individual symptoms and laboratory parameters were calculated. Diagnostic performance was further assessed using univariate and multivariate logistic regression and nomogram analyses.

**Results:**

Among 2,015 children tested, 724 (35.9%) were positive for pertussis, with 187 (30.2%) hospitalized and 55 (8.9%) admitted to the ICU. Cases increased from August 2023, peaking in May 2024, with the majority aged 3–11 years (51.8%). Compared with B. pertussis–negative children, positive cases exhibited higher WBC counts and procalcitonin (PCT) levels. Incorporating PCT into the WHO suspected-case definition improved the ROC area from 0.710 to 0.870, with an optimal cutoff of 0.1665 ng/mL. Cyanosis, post-tussive vomiting, neutrophil count, and assisted ventilation emerged as independent predictors of ICU admission, with a neutrophil cutoff of 6.62 × 10^9^/L.

**Conclusion:**

The age distribution of pediatric pertussis shifted from young infants to preschool- and school-aged children. Clinical features alone are insufficient for reliable diagnosis due to overlap with non-pertussis cases. Elevated neutrophil count serves as an independent predictor of ICU admission, highlighting its potential utility for early risk stratification in pediatric pertussis.

## Introduction

Pertussis (whooping cough), caused by Bordetella pertussis, is an acute respiratory infectious disease characterized primarily by paroxysmal spasmodic coughing (Kilgore et al., 2016; Matthieu and Pejman, 2024). Despite the widespread global use of pertussis vaccines, the incidence of the disease has risen significantly in multiple regions in recent years (Smout et al., 2024; Monchausse et al., 2025). China has similarly experienced this resurgence, with 32,380 cases reported in mainland China in 2024 (National Health Commission of China, 2024), and a concurrent shift in the age distribution of cases (Hu et al., 2024), highlighting the continuing challenges in controlling the disease. It remains unclear whether vaccination practices and changes in the affected age groups have altered the clinical presentation of pertussis (Crowcroft et al., 2003). In this context of widespread immunization, evidence remains limited regarding the diagnostic accuracy—particularly the sensitivity and specificity—of clinical manifestations in identifying pertussis, especially among pediatric patients.

Early case identification is central to pertussis control. The WHO and multiple countries have therefore developed diagnostic criteria for suspected pertussis to guide clinical suspicion, testing, and early detection (WHO-recommended surveillance standard of pertussis, 2019). However, in the context of the recent pertussis resurgence, there is a scarcity of research assessing the diagnostic performance of these various case criteria in children (Muloiwa et al., 2020).

Pertussis patients commonly present with clinical manifestations and symptoms such as paroxysmal spasmodic coughing, whooping, cyanosis, and post-tussive vomiting (Hanna and Samies, 2025). Peripheral white blood cell counts, particularly lymphocyte counts, may be significantly elevated. Patient symptoms and laboratory findings substantially aid clinicians in resource-limited settings for pertussis identification. While PCT and high-sensitivity C-reactive protein (hs-CRP) are commonly used biomarkers for auxiliary diagnosis of infectious diseases, only a few studies have assessed their diagnostic value in pertussis diagnosis (Elhoufey, 2025; Gan and Wu, 2025; Huo et al., 2025). Whether the integration of laboratory parameters with standardized clinical criteria can enhance diagnostic performance remains an open question.

Despite a generally favorable prognosis, pertussis can lead to serious complications such as apnea, severe pneumonia, and encephalopathy. The identification of reliable risk factors for severe disease is of great clinical importance.

This study aims to, first, analyze the epidemiological and clinical characteristics of pediatric pertussis and compare the symptom profiles of *B. pertussis*-positive and -negative children to assess their diagnostic utility; second, evaluate the diagnostic performance of WHO and Chinese clinical criteria, both alone and when augmented with laboratory findings; and third, identify independent risk factors and establish predictive cutoffs for ICU admission. An ancillary aim was to explore the diagnostic value of hs-CRP and PCT. The results are expected to offer scientific evidence to improve diagnostic accuracy and severity assessment in pediatric pertussis.

## Materials and methods

### Study design and population

This retrospective study was conducted at Guangdong Women and Children Hospital, a major tertiary care center in Guangzhou, China. The study aimed to delineate the prevalence trends and comprehensively analyze the clinical and laboratory characteristics of pediatric pertussis. We enrolled patients aged ≤14 years who presented with a primary complaint of cough and underwent pertussis-related laboratory testing at our hospital.

The study protocol was approved by the Medical Ethics Committee of our institution (Approval No. 20251008) and was conducted in accordance with the principles of the Declaration of Helsinki. The requirement for informed consent was waived by the ethics committee due to the retrospective nature of the research.

### Data collection

Demographic characteristics, clinical symptoms, laboratory results, and hospitalization details were extracted from the electronic medical record system. Clinical data included sex, age, occupation, cough duration and pattern, fever, facial flushing, vomiting, and admitting diagnosis. Laboratory parameters consisted of white blood cell (WBC), neutrophil, and lymphocyte counts, as well as high-sensitivity C-reactive protein (hs-CRP) and procalcitonin (PCT) levels. All laboratory data and clinical symptoms in our study are based on the information recorded during the initial visit. Leukocytosis and lymphocytosis were defined as WBC count ≥16×10^9^/L and lymphocyte count ≥9×10^9^/L, respectively. All patient data were anonymized and securely stored to ensure confidentiality.

### *B. pertussis* culture and PCR

Nasopharyngeal swab specimens were collected for *B. pertussis* culture. Samples were directly inoculated onto charcoal agar supplemented with 10% defibrinated sheep blood and cephalexin (OXOID, UK) and incubated at 37°C in a humidified atmosphere for up to 5 days. Cultures were examined daily from day 3 onward. Suspected colonies were confirmed as *B. pertussis* using matrix-assisted laser desorption/ionization time-of-flight mass spectrometry (MALDI-TOF MS; Bruker, USA).

For PCR detection, nucleic acids were extracted from nasopharyngeal swabs or bronchoalveolar lavage fluid and analyzed on an ABI 7500 qPCR system (Applied Biosystems) using the *Bordetella pertussis* Real-time PCR Detection Kit (Da An Gene Co., Ltd.). The product information can be accessed at the following URL: https://www.daangene.com/pt/index163.html. Amplification targeted the IS481 insertion sequence gene and ptxA-pr gene, with results interpreted as follows: positive (Ct ≤38), negative (Ct >38 or no amplification with valid internal control), or invalid (internal control Ct >40 or absent). Detailed information can be found in the kit instructions.

### Grouping strategy

Participants were initially classified into *B. pertussis*-positive [*B. pertussis* (+)] or *B. pertussis*-negative [*B. pertussis* (−)] groups based on laboratory test results for comparative analysis of symptoms and laboratory findings. To assess age-specific variations, both *B. pertussis* (+) and *B. pertussis* (−) groups were further stratified into three subgroups: ≤6 months, 6–12 months, and >12 months. Additionally, *B. pertussis* (+) patients were categorized into ICU and non-ICU groups to identify risk factors for severe disease progression.

### Diagnostic criteria

We applied two clinical case definitions for pertussis:

1. WHO criteria: cough lasting ≥2 weeks, plus at least one of the following: paroxysmal coughing, inspiratory whooping, or post-tussive vomiting.
2. Chinese national criteria: paroxysmal spasmodic cough lasting ≥2 weeks; or in infants, apnea, choking, cyanosis, or bradycardia with epidemiological linkage; or in older individuals, persistent cough >2 weeks without fever plus relevant exposure history.

### Statistical analysis

Statistical analyses and graphing were performed using GraphPad Prism version 10.0. Normally distributed continuous variables were expressed as mean ± standard deviation and compared using Student’s t-test; non-normally distributed data were summarized as median [Q1, Q3] and compared with the Mann–Whitney U test. Categorical variables were presented as frequencies (percentages) and analyzed using the chi-square test or Fisher’s exact test, as appropriate. Univariate and multivariate logistic regression models were employed to identify factors associated with pertussis diagnosis and ICU admission. A two-sided p-value < 0.05 was considered statistically significant.

## Results

**Figure 1:**
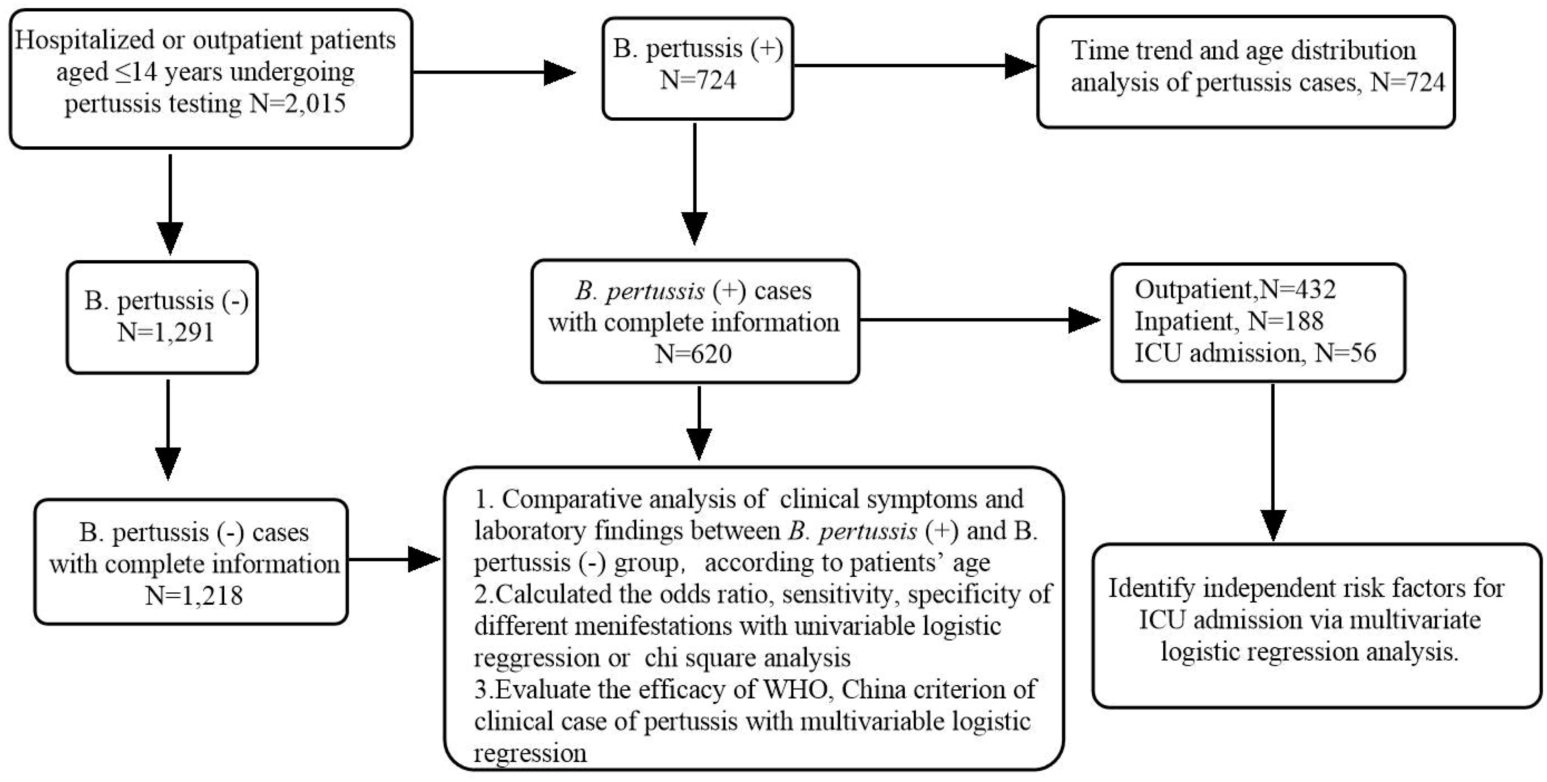
Flowchart of the study.

Between January 2023 and December 2024, a total of 2,015 pediatric patients (aged ≤14 years) presenting with cough as their primary symptom were tested for pertussis at our institution using culture, PCR, or metagenomic sequencing. Among them, 724 (35.93%) tested positive for *B. pertussis* (**Figure 1**). Of the positive cases, 620 (85.63%) had complete medical records available for detailed analysis.

The cohort with complete data comprised 347 (57.83%) men and 273 (42.17%) women. Age stratification revealed that 174 (28.06%) were under 6 months, 30 (4.83%) were aged 6–12 months, and the majority, 416 (67.09%), were over 12 months old.

Notably, 188 patients (30.32%) required hospitalization, with 56 (9.03%) requiring ICU-level care. A strong inverse correlation was observed between age and disease severity: among infants under 6 months, 112 (64.37%) were hospitalized, including 46 (26.44%) admitted to the ICU. In the 6–12-month age group, 12 (40.00%) were hospitalized, with 3 (10.00%) in the ICU. In contrast, among children over 12 months, only 65 (15.38%) required hospitalization, and 7 (1.68%) needed ICU care.

### Time trends and age distribution of pertussis cases

**Figure 2:**
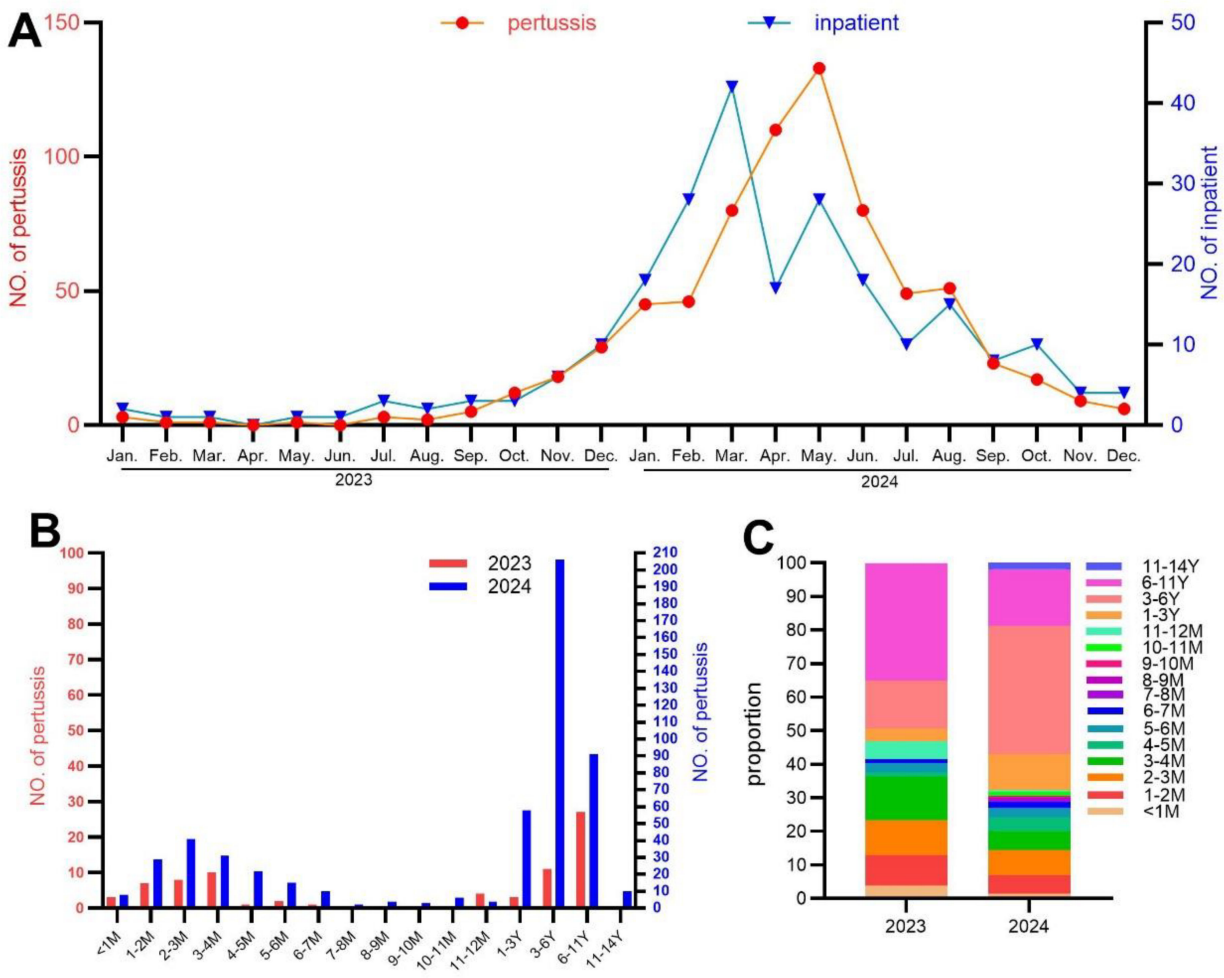
Epidemiological characteristics of pertussis. **(A)** Distribution of pertussis cases (red) and number of patients need hospitalization (blue) across study period. **(B)** Distribution of pertussis cases according to patients’ age (2023−red, 2024−blue). **(C)** Age composition of pertussis cases.

A steady increase in the number of *B. pertussis*-positive cases and associated hospitalizations was observed beginning in August 2023, reaching respective peaks in May and March 2024, after which both trends declined (**Figure 2A**). Analysis of age distribution indicated that *B. pertussis* (+) cases were concentrated primarily among infants aged 1–4 months and children aged 3–11 years (**Figure 2B**). Furthermore, a shift in age distribution was noted between 2023 and 2024: The proportion of school-aged children (6–11 years) with laboratory-confirmed pertussis decreased, whereas the proportion of preschool children (3–6 years) increased (**Figure 2C**).

### Comparisons between *B. pertussis* (+) group and *B. pertussis* (−) group

As shown in **Table 1**, no significant differences were observed in gender distribution between the *B. pertussis* (+) and *B. pertussis* (–) groups across different age strata. In the population aged < 6 months, the proportion of patients requiring hospitalization was significantly higher in the *B. pertussis* (+) group than in the *B. pertussis* (–) group (64.37% *vs*. 46.48%, p = 0.0002), along with a significantly greater proportion requiring ICU admission (26.44% *vs*. 9.15%, p < 0.0001). In contrast, no significant differences were found between the *B. pertussis* (+) and *B. pertussis* (−) groups in terms of hospitalization or ICU admission rates among individuals aged >6 months.

**Table 1:** Comparison of demographic and clinical features of *B. pertussis* (+) and *B. pertussis* (−) groups.

Items	<6M			6-12M			>12M		
	B. pertussis(+) (n=174)	B. pertussis(−) (n=284)	p	B. pertussis(+) (n=30)	B. pertussis(−) (n=126)	p	B. pertussis(+) (n=416)	B. pertussis(−) (n=808)	p
Gender									
Male, N(%)	102(58.62%)	167(58.8%)	>0.9999	17(56.67%)	77(61.11%)	0.6819	228(54.81%)	457(56.56%)	0.5587
Hospitalization status									
Admission yes, N(%)	112(64.37%)	132(46.48%)	0.0002	12(40%)	42(33.33%)	0.5256	64(15.38%)	149(18.44%)	0.1816
ICU admission, N(%)	46(26.44%)	26(9.15%)	<0.0001	3(10%)	3(2.38%)	0.0857	7(1.68%)	8(0.99%)	0.2886
Preliminary diagnosis at admission									
Acute bronchitis, N(%)	16(9.2%)	115(40.49%)	<0.0001	13(43.33%)	48(38.1%)	0.6784	167(40.14%)	421(52.1%)	<0.0001
Bronchopneumonia, N(%)	68(39.08%)	89(31.34%)	0.1046	7(23.33%)	44(34.92%)	0.2815	72(17.31%)	208(27.54%)	0.0009
Upper respiratory tract infection, N(%)	3(1.72%)	17(5.99%)	0.0337	3(10%)	6(4.76%)	0.3758	57(13.7%)	30(3.71%)	<0.0001
Severe pneumonia, N(%)	51(29.31%)	11(3.87%)	<0.0001	3(10%)	2(1.59%)	0.0493	6(1.44%)	4(0.5%)	0.098
Clinical manifestations									
Cough>14 days, N(%)	60(34.48%)	51(17.96%)	<0.0001	6(26.67%)	52(41.27%)	0.0358	122(29.33%)	462(57.18%)	<0.0001
Paroxysmal cough, N(%)	138(79.31%)	147(51.64%)	<0.0001	22(73.33%)	52(41.27%)	0.002	247(59.38%)	313(38.74%)	<0.0001
Inspiratory whooping, N(%)	23(13.22%)	7(2.46%)	<0.0001	5(16.67%)	4(3.17%)	0.0137	19(4.57%)	9(1.11%)	0.0003
Facial flushing, N(%)	94(54.02%)	63(22.18%)	<0.0001	12(40%)	18(14.29%)	0.0014	67(16.11%)	67(8.19%)	<0.0001
Cyanosis, N(%)	55(31.61%)	19(6.69%)	<0.0001	3(10%)	2(1.59%)	0.0493	13(3.13%)	3(0.37%)	0.0001
Post-tussive emesis, N(%)	12(6.9%)	42(14.79%)	0.011	7(23.33%)	39(30.95%)	0.507	89(21.39%)	121(14.98%)	0.0048
Fever, N(%)	134(77.01%)	208(73.24%)	0.3785	19(63.33%)	63(50%)	0.2248	255(61.3%)	455(56.31%)	0.0941
Laboratory findings									
Leukocytosis, N(%)	30(17.24%)	19(6.69%)	0.0004	5(16.67%)	8(6.35%)	0.1322	43(10.34%)	34(4.21%)	<0.0001
Lymphocytosis, N(%)	30(17.24%)	24(8.45%)	0.0046	8(26.67%)	12(9.52%)	0.028	49(11.78%)	33(4.08%)	<0.0001
hsCRP>10 mg/L, N(%)	14(8.05%)	21(7.39%)	0.8568	1(3.33%)	9(7.14%)	0.6882	38(9.13%)	113(13.99%)	0.0168
PCT>0.1 ng/ml, N(%)	154(88.51%)	63(22.18%)	<0.0001	26(86.67%)	5(3.97%)	<0.0001	348(83.65%)	52(6.44%)	<0.0001
Ventilation status									
With assisted ventilation, N(%)	33(18.97%)	15(5.28%)	<0.0001	1(3.33%)	2(1.59%)	0.4755	9(2.16%)	5(0.62%)	0.0222
Non-invasive ventilation, N(%)	18(10.34%)	6(2.11%)	0.0003	1(3.33%)	2(1.59%)	0.4755	7(1.68%)	1(0.12%)	0.0029
Invasive ventilation, N(%)	15(8.62%)	9(3.17%)	0.0161	0	0	/	2(0.48%)	4(0.5%)	>0.9999

Substantial differences in initial diagnoses at the first medical visit were observed between the *B. pertussis* (+) and *B. pertussis* (−) groups across age groups. Among infants < 6 months, the proportion of infants diagnosed with severe pneumonia was significantly higher in the *B. pertussis* (+) group than in the *B. pertussis* (−) group (29.31% *vs*. 3.87%, p < 0.0001). Conversely, diagnoses of acute bronchitis and acute upper respiratory infection were significantly less frequent in the *B. pertussis* (+) group than in the *B. pertussis* (–) group (9.2% *vs*. 40.49%, p < 0.0001; and 1.72% *vs*. 5.99%, p = 0.0337, respectively). No significant difference was observed in the bronchopneumonia diagnosis rate between the two groups in this age stratum. No significant differences in initial diagnoses were observed between the two groups for children aged 6–12 months. Among those aged >12 months, the *B. pertussis* (–) group had higher proportions of patients diagnosed with acute bronchitis and bronchopneumonia (52.21% *vs*. 40.14%, p < 0.0001; and 27.54% *vs*. 17.31%, p = 0.0009, respectively), whereas the proportion of patients diagnosed with acute upper respiratory infection was lower than that in the *B. pertussis* (+) group (13.7% *vs*. 3.71%, p < 0.0001).

The proportions of patients presenting with spasmodic cough, whooping, facial flushing, and cyanosis were significantly higher in the *B. pertussis* (+) group than in the *B. pertussis* (−) group across all age groups. Details are provided in **Table 1**. In infants < 6 months, the proportion with cough duration of >14 days was significantly greater in the *B. pertussis* (+) group than in the *B. pertussis* (−) group (34.48% *vs*. 17.96%, p < 0.0001). In contrast, among those older than 6 months, the proportion of patients with cough lasting >14 days was significantly higher in the *B. pertussis* (−) group. For patients over 12 months of age, post-tussive vomiting was more frequent in the *B. pertussis* (+) group (21.39% *vs*. 14.98%, p < 0.0001). No significant intergroup differences were observed in the proportion of afebrile patients across any age group.

Regarding laboratory findings, the *B. pertussis* (+) group exhibited significantly higher rates of leukocytosis (for the <6M group: 17.24% *vs*. 6.69%, p=0.0004; for 6-12M group: 16.67% *vs*. 6.35%, p=0.1322; for >12M group: 10.34% *vs*. 4.21%, p<0.0001) or lymphocytosis (for<6M group: 17.24% *vs*. 8.45%, p=0.0046; for 6-12M group: 26.67% *vs*. 9.52%, p=0.028; for >12M group: 11.78% *vs*. 4.08%, p<0.0001) than the *B. pertussis* (–) group across all age strata. There were no significant differences between the *B. pertussis* (+) and *B. pertussis* (–) groups in the proportion of patients with hs-CRP >10 mg/L in any age category. However, the proportion of patients with PCT >0.1 ng/mL was significantly higher in the *B. pertussis* (+) group across all age groups (for the <6M group: 88.51% *vs*. 22.18%, p<0.0001; for the 6-12M group: 86.67% *vs*. 3.97%, p<0.0001; for the >12M group: 83.65% *vs*. 6.44%, p<0.0001). The proportion of infants younger than 6 months requiring assisted ventilation was significantly greater in the *B. pertussis* (+) group than in the *B. pertussis* (−) group (18.97% *vs*. 5.25%, p < 0.0001).

**Figure 3:**
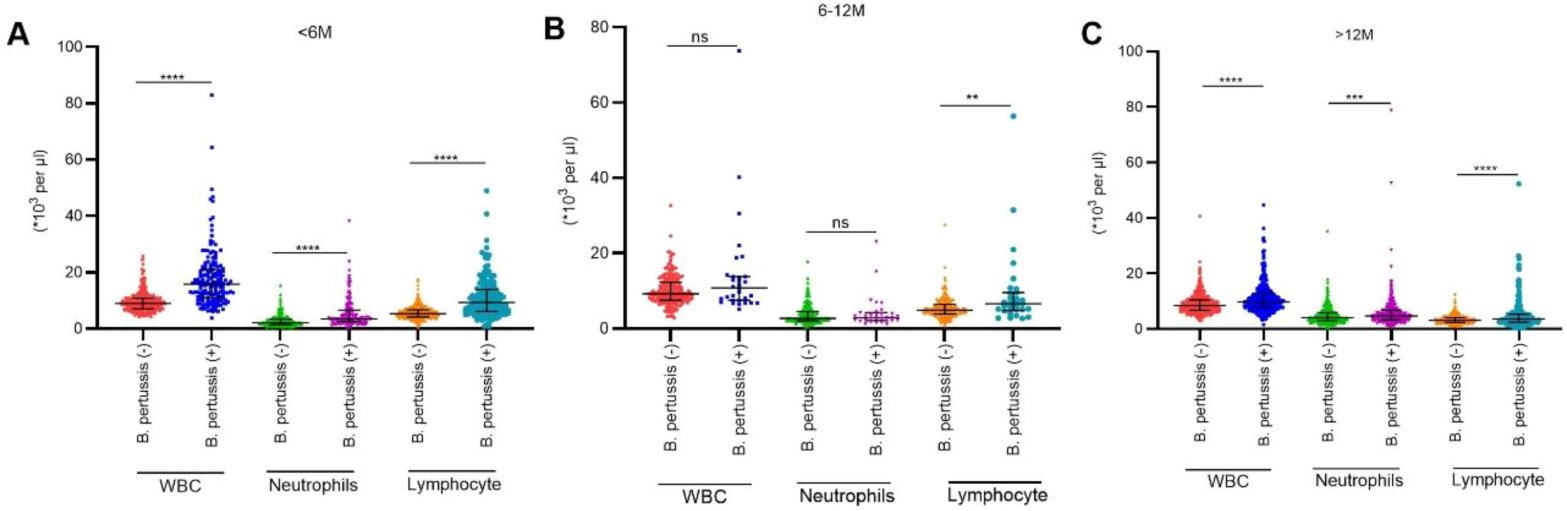
Comparison of peripheral blood cell parameters between *B*. *pertussis* (+) and *B*. *pertussis* (−) groups. **(A)** Comparison of peripheral WBC, neutrophils, and lymphocyte between *B. pertussis* (+) and *B*. *pertussis* (−) groups in infants aged <6 months. **(B)** Comparison of peripheral WBC, neutrophils, and lymphocyte between *B. pertussis* (+) and *B*. *pertussis* (−) groups in infants aged 6–12 months. **(C)** Comparison of peripheral WBC, neutrophils, and lymphocyte between *B. pertussis* (+) and *B*. *pertussis* (−) groups in children aged >12 months. **P<0.05,*** p<0.001,**** p<0.0001.

**Figure 4:**
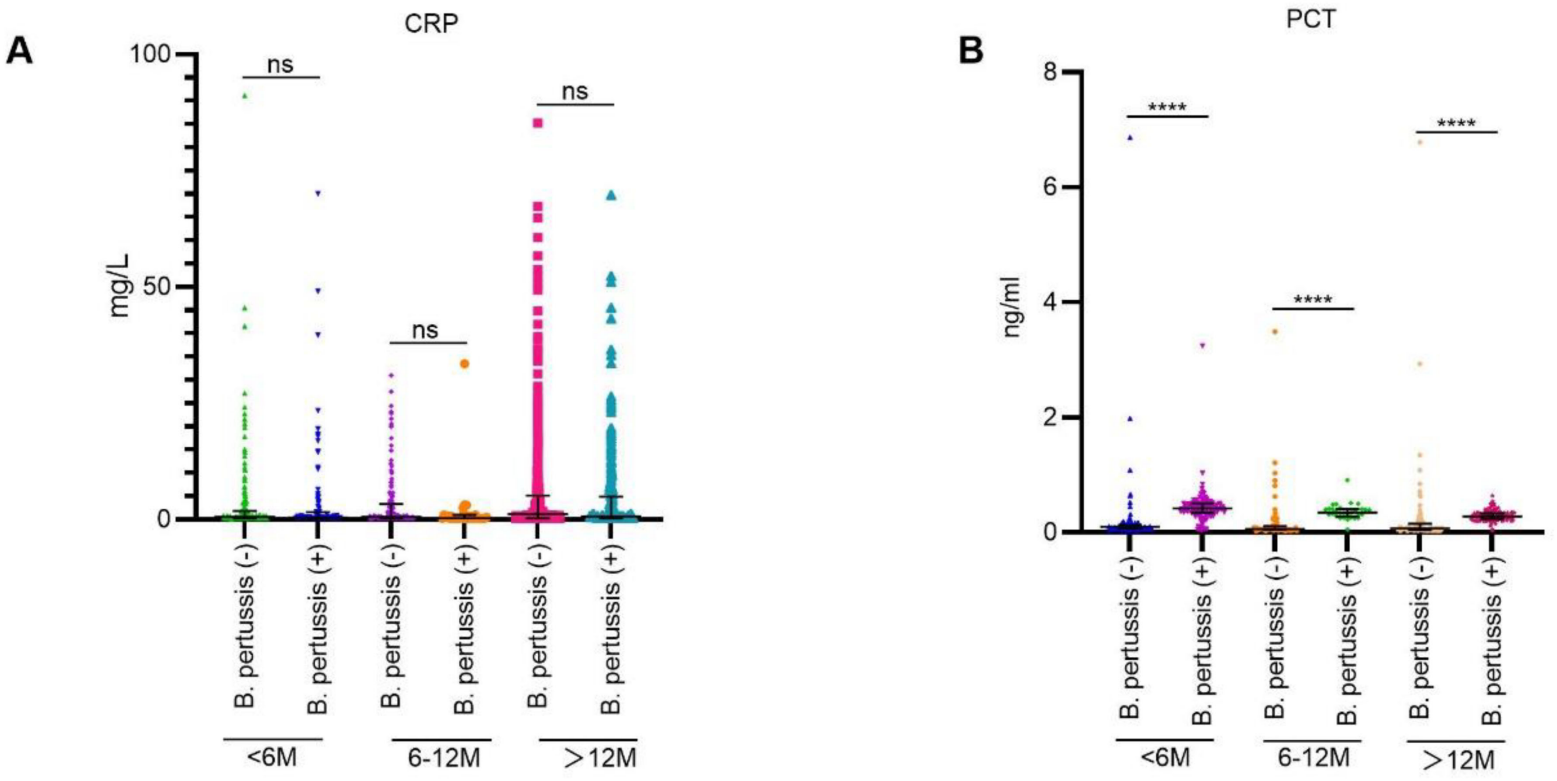
Comparison of inflammatory indicators between *B*. *pertussis* (+) and (*B*) *pertussis* (−) groups. **(A)** Comparison of blood hs-CRP level between *B. pertussis* (+) and *B*. *pertussis* (−) groups in different age groups. **(B)** Comparison of blood PCT level between *B. pertussis* (+) and *B*. *pertussis* (−) groups in different age groups. ****p<0.0001.

As illustrated in **Figure 3**, there were notably higher counts of WBC, neutrophils, and lymphocytes in the *B. pertussis* (+) group compared with the *B. pertussis* (−) group among infants aged <6 months and those aged > 12 months. In the 6–12-month age group, no significant differences were observed in peripheral WBC and neutrophil counts between the *B. pertussis* (+) and *B. pertussis* (−) groups; however, the lymphocyte count was significantly higher in the *B. pertussis* (+) group. As illustrated in **Figure 4**, across all age groups, no significant differences were observed in the blood hs-CRP levels between the *B. pertussis* (+) and *B. pertussis* (−) groups. In contrast, serum PCT levels were significantly elevated in the *B. pertussis* (+) group compared with the *B. pertussis* (−) group.

### The diagnostic efficiency of manifestations and laboratory findings

**Figure 5:**
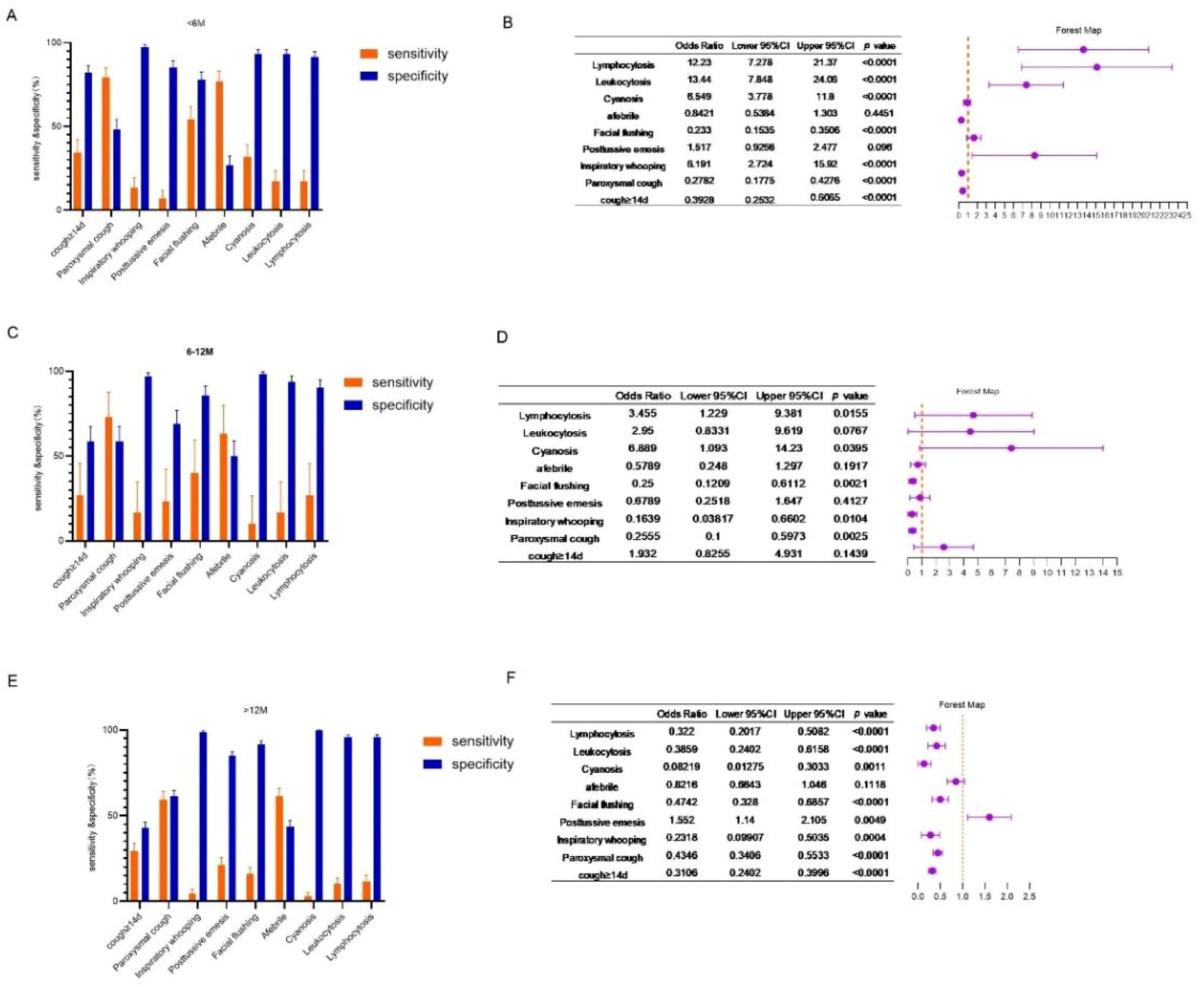
Sensitivity, specificity, and odds ratio of different clinical features in diagnosis of pertussis. **(A)** Sensitivity and specificity of clinical features in diagnosis of pertussis in the <6-month age group. **(B)** Forest map of odds ratio of different clinical features in the aged <6-month age group. **(C)** Sensitivity and specificity of clinical features in diagnosis of pertussis in diagnosis of pertussis in the 6-12-month age group. **(D)** Forest map of odds ratio of different clinical features in the 6-12-month age group. **(E)** Sensitivity and specificity of clinical features in diagnosis of pertussis in the >12-month age group. **(F)** Forest map of odds ratio of different clinical features in the >12-month group.

The sensitivity and specificity of various clinical symptoms and laboratory findings in identifying children with pertussis exhibit considerable variation (**Figure 5**). In infants aged <6 months, spasmodic cough and absence of fever demonstrated high sensitivity but low specificity; facial flushing showed moderate sensitivity and specificity; and whooping, cyanosis, leukocytosis, and lymphocytosis all exhibited high specificity but generally low sensitivity (**Figure 5A**). Univariate logistic regression analysis revealed that whooping, cyanosis, leukocytosis, and lymphocytosis were associated with high odds ratios (ORs) in the <6-month age group, whereas facial flushing, spasmodic cough, and cough duration >14 days were associated with lower ORs (**Figure 5B**).

Spasmodic cough and absence of fever displayed moderate sensitivity and specificity among children aged 6–12 months, whereas whooping, cyanosis, leukocytosis, and lymphocytosis maintained high specificity but generally low sensitivity (**Figure 5C**). Univariate logistic regression indicated that high ORs were associated with cyanosis and lymphocytosis in this age group, whereas low ORs were associated with facial flushing, spasmodic cough, and whooping (**Figure 5D**).

In children aged >12 months, spasmodic cough and absence of fever again demonstrated moderate sensitivity and specificity; whooping, cyanosis, leukocytosis, and lymphocytosis continued to show high specificity but generally low sensitivity (**Figure 5E**). According to univariate logistic regression, post-tussive vomiting was associated with a high OR in the >12-month group, whereas facial flushing, spasmodic cough, cough duration >14 days, whooping, leukocytosis, and lymphocytosis were associated with lower ORs (**Figure 5F**).

### The performance of different suspected pertussis diagnostic criteria alone or combine with other factors

**Figure 6:**
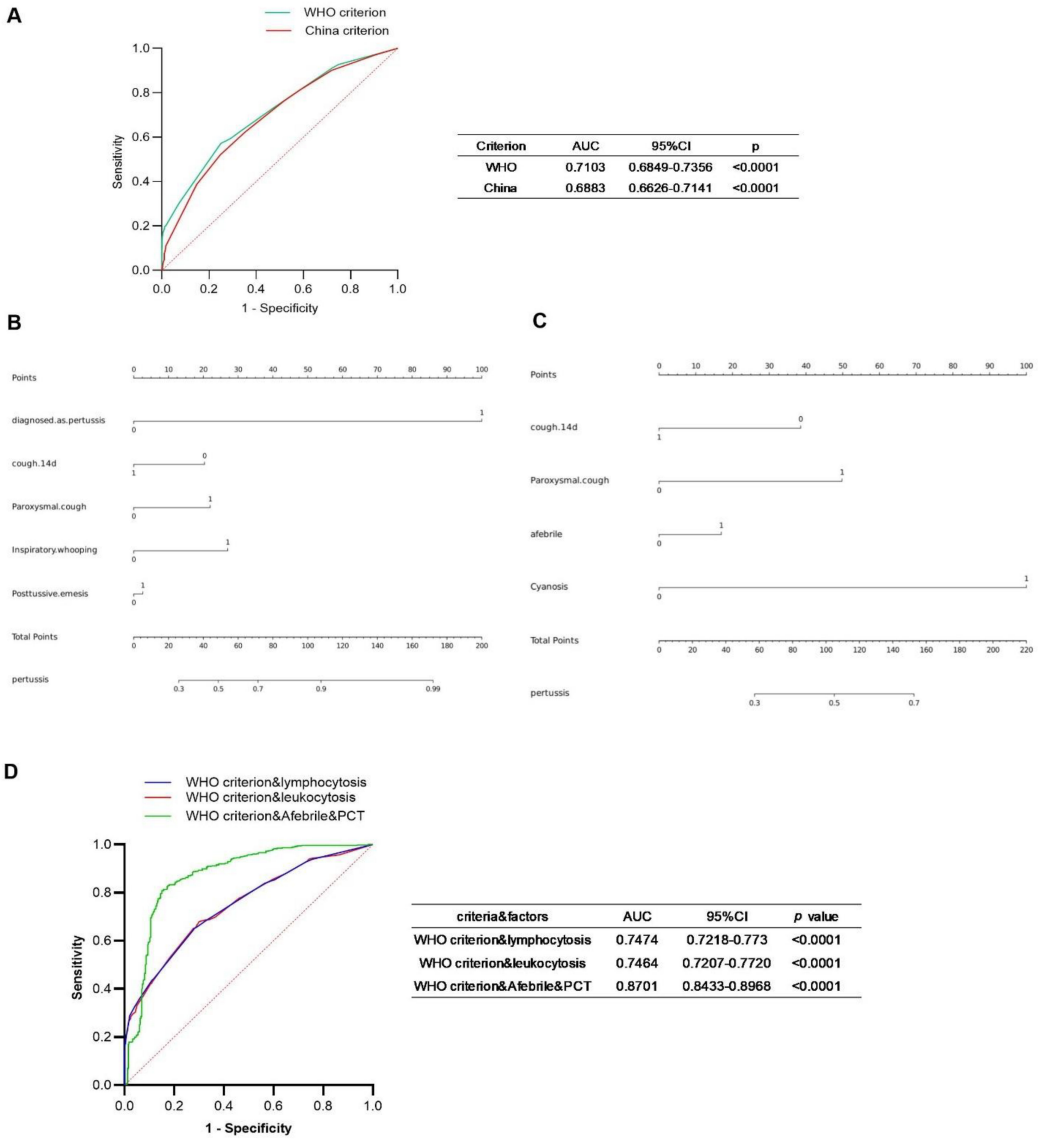
Performance of different criteria alone or in combination with other factors in pertussis diagnosis using multivariable logistic regression. **(A)** ROC curves of WHO or China criterion for clinical diagnosis of pertussis and respective AUC. **(B, C)** Nomogram analysis was performed on symptoms indicated by WHO or China criterion for clinical diagnosis of pertussis. **(D)** Combined ROC curves of WHO criterion-recommended symptoms with other factors as indicated.

ROC analysis was performed separately on the symptom combinations recommended by the WHO and Chinese diagnostic criteria for suspected pertussis cases. The AUC of ROC for the case features recommended by the WHO criteria was 0.7103 (95% CI: 0.6849–0.7356), whereas the AUC for features recommended by the Chinese criteria was 0.6883 (95% CI: 0.6626–0.7141) (**Figure 6A**). Nomogram analysis demonstrated that the predictive efficacy of different symptoms or clinical manifestations in pertussis prediction varied (**Figures 6B, C**).

Further analysis revealed that the combination of the WHO criteria with elevated leukocytosis or lymphocytosis significantly increased the AUC to 0.7464 (95% CI: 0.7207–0.772) or 0.7474 (95% CI: 0.7218–0.773), respectively. Combining the WHO criteria with patient fever status and PCT levels yielded a further substantial increase in AUC, reaching 0.8701 (95% CI: 0.8433–0.8968) (**Figure 6D**).

**Figure 7:**
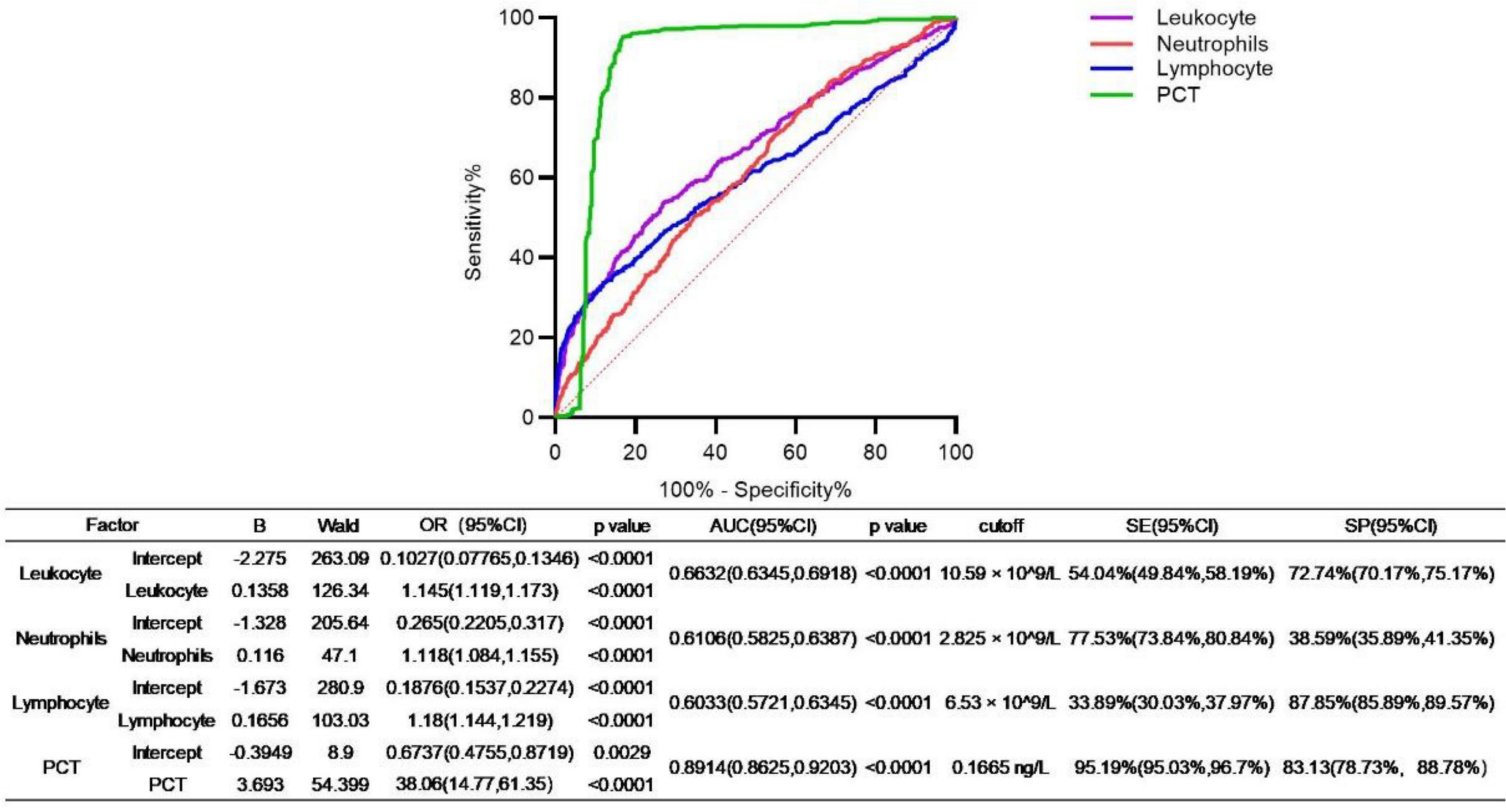
Univariable logistic regression of leukocyte, neutrophils, lymphocyte, and PCT. The ROC of factors that increased the performance of the WHO criterion. The OR, AUC, and optimal cutoff value as well as related sensitivity (SE) or specificity (SP) of the indicated factors were presented in the table.

Given that leukocytosis, lymphocytosis, and PCT level each significantly increased the AUC of WHO criteria, we further performed univariate logistic regression analyses to evaluate the diagnostic performance of these indicators. Additionally, we determined their optimal cutoff values for predicting pertussis, along with the corresponding sensitivity and specificity. As shown in **Figure 7**, the OR of leukocyte was 1.145 (95% CI: 1.119–1.173), and the AUC was 0.6632 (95% CI: 0.6345–0.6918), and the optimal cutoff point for leukocyte was 10.59 × 10^9^/L, with a sensitivity of 54.04% (95% CI: 49.84%–58.19%) and a specificity of 72.74% (95% CI: 70.17%–75.17%). The OR of neutrophil was 1.118 (95% CI: 1.084–1.155), and the AUC was 0.6106 (95% CI: 0.5825–0.6387), the optimal cutoff point for neutrophil was 2.285 × 10^9^/L, with a sensitivity of 77.53% (95% CI: 73.84%–80.84%) and a specificity of 38.59% (95% CI: 35.89%–41.35%). The OR of lymphocyte was 1.180 (95% CI: 1.144–1.219), and the AUC was 0.6033 (95% CI: 0.5721–0.6345), the optimal cutoff point for lymphocyte was 6.53 × 10^9^/L, with a sensitivity of 33.89% (95% CI: 30.03%–37.97%) and a specificity of 87.85% (95% CI: 85.89%–89.57%). The OR of PCT was 38.06 (95% CI: 14.77–61.35), and the AUC was 0.8914 (95% CI: 0.8625–0.9203), and the optimal cutoff point for PCT was 0.1665 ng/L, with a sensitivity of 95.19% (95% CI: 93.03%–96.70%) and a specificity of 83.13% (95% CI: 78.73%–88.78%).

### Risk factors for ICU admission

**Figure 8:**
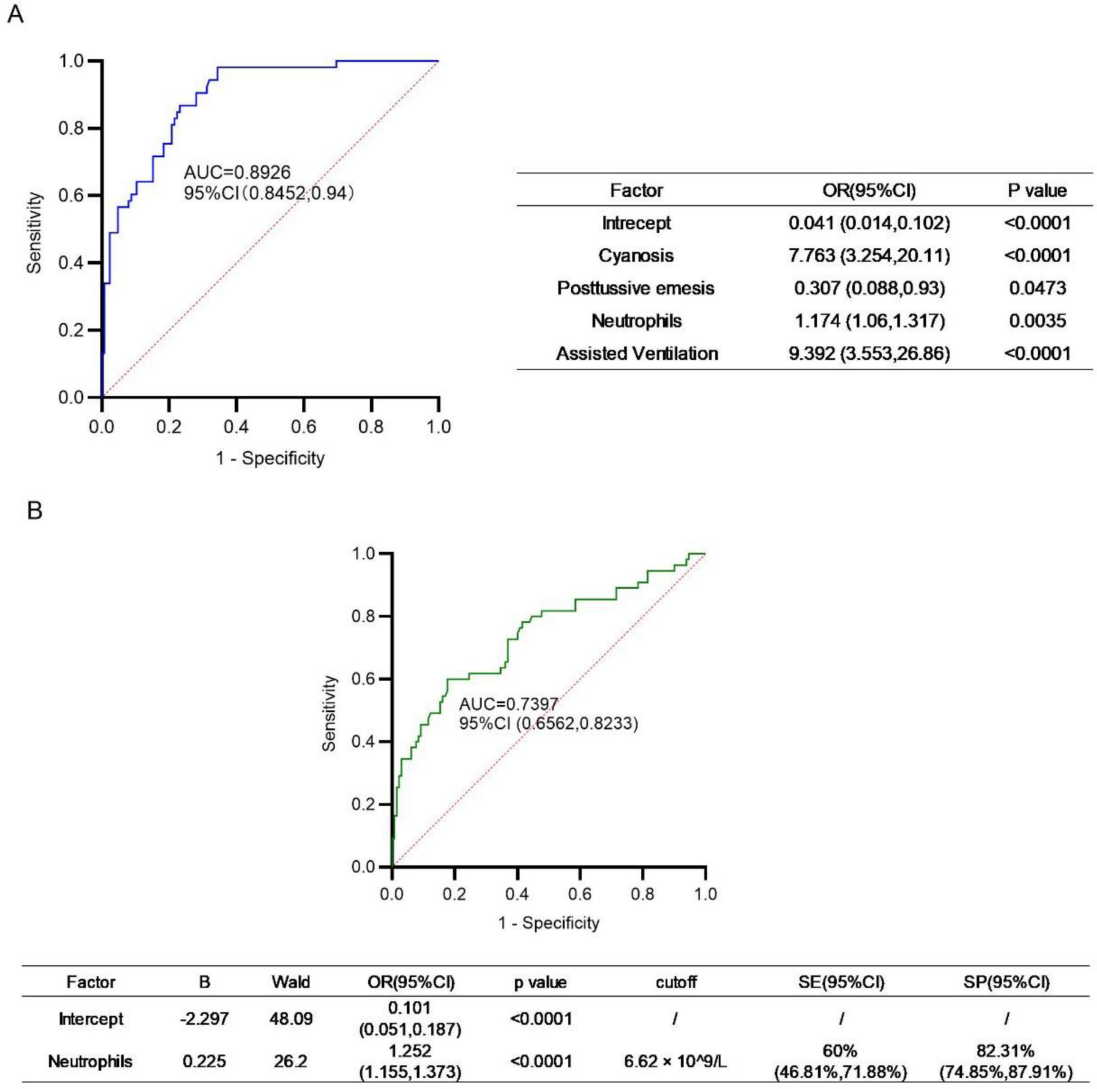
Multivariable logistic regression and univariable logistic regression of indicated factors. **(A)** Combined ROC of independent risk factors associated with ICU admission and their OR. **(B)** ROC of neutrophils in prediction of ICU admission, and the optimal cutoff value as well as related sensitivity (SE) or specificity (SP) of the indicated factors were presented in the table.

Multivariable logistic regression analysis revealed that the independent risk factors for ICU admission in children with pertussis included cyanosis (OR = 7.763, 95% CI 3.254-20.11, *p* < 0.0001), post-tussive vomiting (OR = 0.307, 95% CI 0.088-0.93, *p* = 0.0473), elevated peripheral blood neutrophil count (OR = 1.174, 95% CI 1.06-1.317, *p* = 0.0035), and assisted ventilation (OR = 9.392, 95% CI 3.553-26.86, *p* < 0.0001). The AUC of combined ROC of these factors was 0.8926 (95% CI 0.8452-0.94) (**Figure 8A**). The optimal cutoff value of peripheral neutrophil count for predicting ICU admission in children with pertussis was 6.62 × 10^9^/L, with a sensitivity of 60% (95% CI 46.81%-71.88%) and a specificity of 82.31% (95% CI 74.85%–87.91%) (**Figure 8B**).

## Discussion

As a highly contagious disease, pertussis represents an ongoing challenge to global health, with unvaccinated infants being particularly vulnerable to severe outcomes (Matthieu and Pejman, 2024; Smout et al., 2024; Elhoufey, 2025). The recent changes in its epidemiological characteristics and clinical manifestations (Hu et al., 2024) have prompted renewed scientific inquiry. A detailed analysis of contemporary patient profiles, coupled with an assessment of the efficacy of various diagnostic criteria, is therefore warranted to generate robust scientific evidence for clinical practice.

In accordance with the trends of pertussis across other parts of China (Shi et al., 2024; Liu et al., 2025; Tang et al., 2025), we saw a significant increase in the number of pertussis cases starting in September 2023 (**Figure 2A**). Previously, heightened awareness and changes in diagnostic criteria were considered responsible for the increased pertussis incidence (National Health Commission, 2023). Pertussis-related PCR detection and culture had been performed in our institute for years, which supports a true increase in incidence rather than improved awareness and detection. An upward trend of pertussis cases and a change in the characteristics of pertussis epidemiology during late 2023 and early 2024 were also observed in other countries (Guo et al., 2024; Programme WHE, 2024; Toizumi et al., 2024; Liu et al., 2025; Scutari et al., 2025), suggesting that the trends in China may be part of a wider regional pattern.

Our data indicate that preschool and school-aged children constituted the majority of pertussis cases (**Figure 2B, C**), corroborating the ongoing age shift noted in recent studies (Monchausse et al., 2025). The accumulation of susceptible children after the COVID-19 pandemic is a plausible explanation for this trend. Furthermore, the global pertussis resurgence has been partially driven by genotypic changes in *B. pertussis*. The dominant strain in our center, ptxP3, matches the genotype reported in other Chinese studies (Cai et al., 2023; Li et al., 2025; Mai et al., 2025) and has similarly displaced ptxP1 in the USA and Europe (Bart et al., 2014). This genotypic shift, characterized by adaptive mutations in antigen genes, is believed to enhance the strain’s fitness and transmissibility (Cai et al., 2023).

Clinical features such as paroxysmal coughing, inspiratory whoop, post-tussive vomiting, and facial flushing were more prevalent in *B. pertussis* (+) cases. However, a considerable proportion of patients in the *B. pertussis* (−) group with cough as the primary symptom also exhibited these manifestations (**Table 1**). Generally, the sensitivity and specificity of individual symptoms or clinical signs for identifying pertussis cases are low (**Figure 5**), suggesting that reliance on any single clinical feature is insufficient for a precise diagnosis. These findings align with those of prior literature (Muloiwa et al., 2020). The widespread occurrence of paroxysmal coughing and similar symptoms in cases not caused by pertussis may be linked to shifts in the pattern of respiratory pathogens following the COVID-19 pandemic. Following the pandemic, mainland China saw a considerable surge in *Mycoplasma pneumoniae* and *Chlamydia pneumoniae* infections (Lan et al., 2024; Yang et al., 2025), which coincided with a renewed pertussis outbreak. Notably, *M. pneumoniae* and *Chlamydia pneumoniae* infections often present with a prolonged or paroxysmal cough as key clinical features (Zhu et al., 2024). During the paroxysmal stage of whooping cough, the sensitivity of both culture and PCR testing declines significantly (Riffelmann et al., 2005), increasing the possibility of false-negative results. All these may partially explain the relatively high prevalence of pertussis-like symptoms in subjects who tested negative for pertussis and low OR of cough duration >14 days in identifying pertussis.

Current guidelines, including those from WHO or China, do not consider fever as a typical feature of pertussis, stating the absence or mild presence of fever in these criteria. In our cohort, fever was reported in approximately 30% of cases. Other studies have also found that fever is common among patients with pertussis (Muloiwa et al., 2020; Hughes et al., 2025). Change in epidemiological trends, particularly shifts in the age distribution of infected individuals, may be part of the reason for the observed increase in fever among pertussis patients. A French study reported fever in >50% of pertussis patients aged <1 year, compared with 20% in older children (Hughes et al., 2025), and more than half of pertussis patients aged <1 year in our study (**Figures 2B, C**). Comparable fever rates were observed between *B. pertussis* (+) and *B. pertussis* (−) cases in our study. Although further investigation is warranted, we speculated that the coinfection pathogen other than *B. pertussis* caused the fever. A study conducted in China suggested increased fever incidence among pertussis patients post-COVID-19 pandemic, and during that period, there was an outbreak of *Mycoplasma pneumoniae* and *Chlamydia pneumoniae* in mainland China (Lan et al., 2024; Zhu et al., 2024; Yang et al., 2025), and fever is prevalent among *Mycoplasma pneumoniae* and *Chlamydia pneumoniae* patients. An investigation about coinfection patterns of pertussis is warranted for further explaining the observed phenomenon.

Leukocytosis is a well-documented laboratory abnormality in pertussis (Carbonetti, 2016), often guiding the diagnosis in resource-limited settings. However, only 20% of our patients exhibited leukocytosis (defined as WBC ≥16×10^9^/L) and 22% exhibited lymphocytosis (lymphocyte count ≥9×10^9^/L), contrasting with a recently published paper reporting more than 60% prevalence of both (Scutari et al., 2025). The discrepancy may stem from differing diagnostic thresholds or, more crucially, distinct age distributions across study populations. In our cohort, 28% of patients were infants under 6 months—a subgroup that demonstrated significantly higher WBC and lymphocyte counts than older children or *B. pertussis* (−) controls (**Figure 3**). This is consistent with reports from cohorts with a high proportion of young infants, such as an Iranian study where 60% of participants were infants and the rate of lymphocytosis reached approximately 80% (Shojaei et al., 2014).

Regarding inflammatory markers, hsCRP ≥10 mg/L was less frequent in *B. pertussis* (+) individuals than *B. pertussis* (−) individuals, whereas PCT ≥0.1 ng/mL was more prevalent. Age-stratified analysis revealed no significant hsCRP differences between the *B. pertussis* (+) group and the *B. pertussis* (−) group (**Figure 4A**), consistent with Huang et al. and Wu et al (Wu et al., 2019; Huang et al., 2024), although some studies report higher CRP in pertussis (Elhoufey, 2025). These inconsistencies may reflect variations in the age distribution or coinfection status. A Chinese study highlighted CRP variability based on coinfecting pathogens (Gan and Wu, 2025), with elevated CRP potentially indicating poorer prognosis (Huo et al., 2025).

Study about the usage of PCT in the diagnosis of pertussis is rare (Tascini et al., 2019; Huo et al., 2025). In our study, pertussis patients showed higher levels of PCT than age-matched controls, and declined with age in pertussis cases (**Figure 4B**). Differences in coinfection status may influence PCT levels (Gan and Wu, 2025), and elevated PCT level was correlated with adverse outcomes (Huo et al., 2025). Combining PCT with WHO clinical criteria significantly improved the AUC (**Figure 6D**). Univariate logistic regression yielded an AUC of 0.89 for PCT; at a cutoff of 0.1665 ng/mL, the sensitivity and specificity were 95% and 83%, respectively. Combining WBC, neutrophil, and lymphocyte counts with clinical criteria slightly improved diagnostic accuracy; however, their performance metrics were less effective than PCT, highlighting PCT’s potential as a reliable additional biomarker for pertussis diagnosis.

Early and accurate identification of severe pertussis is critical for reducing complications and mortality (Guo et al., 2024). We identified independent risk factors associated with ICU admission in pertussis patients, including cyanosis, peripheral neutrophil count, requirement for assisted ventilation and post-tussive vomiting. Other than post-tussive vomiting, other factors were positively correlated with ICU admission. Although prior studies suggest that leukocytosis and lymphocytosis are predictive factors for severe pertussis, our data indicate that neutrophil count—rather than WBC or lymphocyte count—was a robust predictor of ICU admission. This aligns with a mortality-based study reporting higher neutrophil counts in fatal pertussis cases (Huo et al., 2025). Given the accessibility and cost-effectiveness of complete blood counts, even in resource-limited settings, we further evaluated the diagnostic performance of the neutrophil count for severe pertussis. At a cutoff of 6.62×10^9^/L, neutrophil count predicted ICU admission with 60% sensitivity and 82.3% specificity.

This study has several limitations that should be considered. First, its retrospective nature resulted in incomplete clinical and laboratory data for some participants, particularly regarding hs-CRP and PCT measurements, which may introduce selection bias. Second, the single-center design may limit the generalizability of our findings, and external validation through multicenter studies is warranted. Future prospective, multicenter investigations will be essential to overcome these limitations and provide more robust evidence for optimizing pertussis management.

## Data Availability

The raw data supporting the conclusions of this article will be made available by the authors, without undue reservation.
